# Making psychiatry moral again: the role of psychiatry in patient moral development

**DOI:** 10.1136/jme-2022-108442

**Published:** 2022-08-19

**Authors:** Doug McConnell, Matthew Broome, Julian Savulescu

**Affiliations:** 1 Oxford Uehiro Centre for Practical Ethics, Faculty of Philosophy, University of Oxford, Oxford, UK; 2 School of Psychology, University of Birmingham, Birmingham, UK; 3 Murdoch Childrens' Research Institute, Melbourne, Victoria, Australia; 4 Melbourne Law School, University of Melbourne, Melbourne, Victoria, Australia

**Keywords:** Psychiatry, Paternalism, Coercion, Morals, Ethics- Medical

## Abstract

Psychiatric involvement in patient morality is controversial. If psychiatrists are tasked with shaping patient morality, the coercive potential of psychiatry is increased, treatment may be unfairly administered on the basis of patients’ moral beliefs rather than medical need, moral disputes could damage the therapeutic relationship and, in any case, we are often uncertain or conflicted about what is morally right. Yet, there is also a strong case for the view that psychiatry often works through improving patient morality and, therefore, should aim to do so. Our goal is to offer a practical and ethical path through this conflict. We argue that the default psychiatric approach to patient morality should be procedural, whereby patients are helped to express their own moral beliefs. Such a procedural approach avoids the brunt of objections to psychiatric involvement in patient morality. However, in a small subset of cases where patients’ moral beliefs are sufficiently distorted or underdeveloped, we claim that psychiatrists should move to a substantive approach and shape the *content* of those beliefs when they are relevant to psychiatric outcomes. The substantive approach is prone to the above objections but we argue it is nevertheless justified in this subset of cases.

## Introduction

Several considerations discourage psychiatric intervention in patient morality. Perhaps most importantly, it recalls historic cases of psychiatric abuse where psychiatrists wielding excessive power coercively treated people, sometimes in pursuit of implausible moral ideals.^1 2^ In the Soviet Union, for example, opposition to the sociopolitical system was seen as a symptom of schizophrenia justifying incarceration. Despite this history, Pearce and Pickard have argued that psychiatry should explicitly aim to improve patient morality.^3^

We present the arguments for and against psychiatric intervention in patient morality, then assess whether this dispute can be resolved with a *procedural* approach whereby psychiatry facilitates expression of *the patient’s* conception of morality. We show that, although a procedural approach avoids most objections to psychiatric intervention in patient morality, it leaves the psychiatrist unable to effectively intervene in cases where patients have seriously distorted or underdeveloped moral views. We argue that this subset of cases justifies a *substantive* approach, whereby psychiatrists address the *content* of patients’ moral views. We conclude that psychiatrists should take the procedural approach by default but move to a substantive approach in the subset of cases where it is necessary to pursue psychiatric goals. i

## Arguments against psychiatric intervention in patient morality

There are several arguments that, implicitly or explicitly, underpin psychiatrists’ reluctance to concern themselves with patient morality. First, many believe that psychiatry should not be used to exert social control or, at least, that this role should be tightly restricted lest it be misused by psychiatrists or co-opted by the state. If we allow psychiatrists to explicitly address patient morality, this will medicalise a huge range of behaviour, massively increasing the potential social control psychiatry can exert.^4^

One version of this objection sees psychiatry as a value-neutral, biomedical endeavour, whereby all normative goals are out of bounds, so interfering with a patient’s morality is psychiatric abuse by definition. Given the problems bioreductionism faces, not just in psychiatry but in medicine more broadly,^5^ we will not pursue that version of the objection here. A more mainstream form of the objection holds that mental disorders have a normative dimension^6^ but distinguishes mental disorders, where treatment may be justified, from social deviance, where treatment is unjustified.^7^ Immorality is deviance, not disorder, and so outside the purview of psychiatry. In any case, exposing a person to psychiatric power because *others* demand that person change (morally or otherwise) cannot be justified. Such an approach is incompatible with the psychiatrist’s role as a fiduciary and goes against the view that treatment must be consented to and in the patient’s best interests. The exception to this is where mentally disordered patients may be detained and forcibly treated due to the risk they pose to others, for example, under the Mental Health Act in England and Wales. But the goal here is to minimise harm, not change patients’ moral views.^8^

Second, even if a modicum of social control is within the remit of psychiatry, it would be wrong to instil any particular conception of morality because we cannot say which conception is correct. This argument comes in two forms. Moral relativists might think that psychiatrists should avoid instilling morality because there are no culture-independent moral truths. We will not pursue that metaethical debate here, but note in passing that moral relativism comes with serious costs, such as being unable to claim that slavery or child abuse are objectively wrong. A different form of the argument holds that we can know some moral truths, but epistemic humility requires us to accept that, in many cases, multiple conflicting moral positions might be true, for example, concerning the permissibility of abortion. Since psychiatrists are unlikely to have greater moral insight than non-psychiatrists, they should not push their moral views on others.

Third, many take an egalitarian view of healthcare, whereby patient morality is irrelevant to the care one should receive. This remains the case even when patients are morally responsible for needing care, for example, the injured terrorist and his innocent victims should be treated equally from a medical standpoint.^9^ However, in this case, the primary concern is not that patients judged morally ‘bad’ will be unfairly denied treatment but that they will be unfairly exposed to treatment.

Finally, if psychiatrists challenge patients’ moral beliefs, that will tend to undermine the therapeutic relationship and thus be self-defeating. Patients will be less likely to share morally charged information relevant to diagnosis and treatment if they fear moral condemnation. Furthermore, given the importance that people attribute to their moral beliefs,^10^ patients will tend to react oppositionally if the psychiatrist challenges those beliefs.

## Arguments for psychiatric intervention in patient morality

Despite the above arguments, Pearce and Pickard^3^ claim that psychiatry routinely works by improving patient morality and should aim to do so. Their view is based on the observation that a wide range of mental disorders have a moral aspect. Some kinds of mental disorders are partly *defined* by dispositions that undermine morality. Borderline personality disorder, for example, can involve frustration and impulsivity while antisocial personality disorder can involve lying and violence. Similarly, some paraphilic disorders are defined by sexual desires that risk harm to non-consenting others, notably children. Moral norm-breaking can also *causally contribute* to mental disorders. In some cases of addiction, for example, people act immorally to secure a drug supply and then feel the need to use drugs to deal with shame and guilt.^11^ Finally, although it is rare, morally unacceptable behaviour is sometimes *symptomatic* of mental disorders. A man with paranoid schizophrenia, for example, might easily become violent while psychotic. Although antipsychotics effectively prevent his symptoms, the patient is ambivalent about maintaining his medication, so the re-emergence of his morally concerning symptoms remains a risk requiring psychiatric attention.

To the extent that moral norm-breaking defines, causes, or is an unavoidable symptom of mental disorders, treatments that support moral growth will be helpful and, as it happens, many routine forms of psychiatric treatment do support such growth.^3 12^ Methadone reduces the appeal of heroin, so that an addicted person who stole to fund their habit is less likely to return to that lifestyle^13^; antidepressants can help a person feel able to meet their obligations to others^14^; attention deficit–hyperactivity disorder (ADHD) medications may reduce recidivism^15^ and so on.

Psychological interventions also support moral agency. Take the following case modified from Pearce and Pickard^3^ :

A man has been under psychiatric care for depression and alcohol misuse following police involvement when he harassed an ex-girlfriend. As part of his presentation, he had become preoccupied with the idea that his former partner was unfaithful to him. These ideas are of non-delusional intensity and he has been referred to therapy because of the anger and jealously he feels towards his ex-partner but regards his violent outbursts towards her as justified and inconsequential. During therapy, he reflects upon his childhood experience of his father’s angry outbursts and becomes aware that his outbursts negatively affect his young son. He begins to empathically identify with his son, thus developing his *capacity* to act morally around his son. His new beliefs about his son’s experience also form the basis for a new moral *motivation* to treat his son better.

New moral capacities and motivations developed in therapy increase the likelihood of moral behaviour and can be built on.

An argument for even broader psychiatric involvement in patient morality comes into focus when we recognise the relationship between morality and flourishing. On a subjective view of flourishing (we use a subjective view for ease of exposition but see Hurka^16^), each person flourishes to the extent that they express their own conception of a good life, that is, their set of hierarchically ordered values such as career, relationships, health and hobbies. Crucially, nearly all people place morality among these values and believe themselves to be essentially moral^10^ so, for most, a degree of moral development is necessary for flourishing. It is increasingly recognised that psychiatry should go beyond treating mental disorder to help people develop and maintain *good* mental health, that is, higher states of flourishing than mere absence of mental disorder.^17^ If most people need to express morality to achieve good mental health, psychiatrists can help by using psychiatric interventions to enhance moral agency, for example, providing guidance for parents with personality disorders^18^ or making medication available to children with ADHD.^19^ Psychiatric promotion of flourishing should, however, be limited by distributive justice. On a prioritarian view of distributive justice, for example, the needs of people with serious mental disorders take priority over the flourishing of the mentally healthy, so resources should only be available for the latter when the needs of the former have been sufficiently met.

What about cases where patients might harm others? Psychiatrists’ risk assessments implicitly judge whether patients lack or are likely to lose, the minimal moral agency required for public safety. The patient’s moral beliefs and capacities influence the risk they pose, for example, whether they can recognise and respond to the moral reasons for maintaining medication, manage emotionally challenging situations, such as a court case, regulate aggressive impulses or appropriately respond to displays of fear or pain. This then informs risk management strategies—the more fragile the patient’s moral agency the greater the justification for intervention. Of course, sometimes such judgements have to be made quickly with partial information, such as when a person is brought to a Place of Safety by the police under Section 136 of the English Mental Health Act, but this just means that the psychiatrist must work with a generalised conception of patient moral agency, not ignore patient moral agency altogether.

In summary, successful psychiatric interventions often depend on implicit assessment of and support for patient morality, and psychiatry would likely be more effective if this moral focus was made explicit. But, if this is right, how can it be squared with the above arguments against psychiatric involvement in patient morality?

## Procedural versus substantive approaches

One way to advance this debate is to make a familiar philosophical distinction between the procedural and the substantive. Those against psychiatric involvement in patient morality are typically thinking in substantive terms, that is, shaping the *content* of patients’ moral beliefs. Conversely, those in favour of psychiatry improving patient morality are typically thinking in procedural terms, that is, facilitating patients’ expression of *their own* morality, whatever its content.

The above case for psychiatric involvement in patient morality is based on the procedural approach. The jealous father’s therapist does not directly challenge the father’s belief that his violent outbursts are inconsequential, but takes an established belief—that he was treated badly by his own father—and helps him see the relevance for parenting his own son. The patient then realises that his violent outbursts harm his son and he revises his moral beliefs accordingly. In the examples where pharmacotherapy supports moral agency, it does so by helping people act in accord with their existing moral beliefs more consistently, for example, helping the addicted person resist temptation and maintain their recovery.

The case against improving patient morality almost entirely dissolves on the procedural approach. There is little concern about coercion or breaching fiduciary duties if the psychiatrist is encouraging the patient to follow the patient’s own moral norms in pursuit of psychiatric goals. Of course, sometimes inconsistency in moral views makes it difficult to know which are truly the patient’s own. A person struggling with addiction, for example, might suddenly relax their moral standards in what might be an attempt to rationalise selfish behaviour at the beginning of a potential relapse. In such cases, the procedural approach is to get the patient to confront and resolve their inconsistency, that is, support the patient in settling on their genuine moral values. This is not coercive as long as the psychiatrist remains neutral on how the inconsistency is resolved.

The therapeutic relationship is not undermined by moral disagreement on the procedural approach because the psychiatrist does not challenge, let alone condemn, the patient’s view of morality. Nevertheless, other kinds of conflict may develop because, *inter alia*, the patient might find it distressing or annoying to resolve inconsistency between moral values or reflect on past failures to meet their own moral standards. If psychiatrists judge that such reflection would be therapeutic, they can motivate it in a non-oppositional way by appealing to the patient’s values. For example, the value of being seen as a rational, morally responsible agent capable of moral growth could encourage reluctant patients to resolve inconsistencies and reflect on past moral failures. Finally, the procedural approach entails treating all patients equally because the psychiatrist can only address morality if it is therapeutically relevant and to the extent that the patient is prepared to discuss it.

If the procedural approach was sufficient, then the problem of patient morality in psychiatry would be more or less resolved. There is, however, a case to be made that psychiatry should sometimes concern itself with patients’ substantive morality.

## Argument for a (limited) substantive approach

When patients have a sufficiently distorted or underdeveloped conception of morality, the psychiatrist should address the content of that conception when it is relevant to psychiatric goals. Consider, first, a case of distorted morality:

A man who grew up in gangs and used to be an ‘enforcer’ justifies his past violence by appealing to the gang’s code of honour and shows no remorse. Later, in his mid-20s, when undertaking a custodial sentence in prison, he develop a psychotic episode and seriously assaults a fellow inmate. Although he has now left the gang and his psychosis is in remission, he continues to believe that violence is justified to ensure respect. He doesn’t believe it is a problem that some people are shown less respect simply because they are unable or unprepared to intimidate others. At psychiatric follow-up upon release from prison, the psychiatrist is concerned that the patient’s moral views increase his risk of violent altercations and further interactions with the criminal justice system which increase his risk of relapse. Furthermore, if he does relapse, his moral views amplify the risk he poses to others.

Beginning with the procedural approach, the psychiatrist might explore the consistency of the patient’s moral views—if what the patient says is correct, then the patient himself will be worthy of less respect when he is older and weaker; this appears to contradict the value he places on being respected. In this case, however, we can imagine that the patient resolves the apparent inconsistency by accepting that losing respect is just another downside of ageing.

When the patient’s moral beliefs are wrong but internally consistent, the procedural approach is unable to facilitate moral growth. This motivates the substantive approach, where the psychiatrist tries to convince the patient to change the *content* of their moral beliefs. In the ex-enforcer’s case, the psychiatrist might try to get him to see that violence is a disproportionate response to disrespect and that morality requires us to respect all people equally, independent of their physical strength. The substantive approach might often require convincing the patient that they are, or can become a valued member of society, since this motivates an interest in maintaining shared moral standards.^ii^

The above objections, however, are now brought back into play: substantive interventions appear to breach fiduciary duty, risk coercively changing patients’ moral views, damage the therapeutic relationship, and psychiatrists’ moral views might be no better than those of the patient. We might intuitively agree that the ex-enforcer’s morality needs substantive attention, but how can we make a principled distinction between such cases and others, where substantive intervention would be unjustified.

One way to set a principled threshold for substantive intervention is to appeal to Rawlsian reasonableness.^21^ Roughly, reasonable moral views treat others as free and equal, ground fair reciprocity and tolerate different moral views where they too are reasonable. Unreasonable moral views, on the other hand, do not treat others as free and equal, ground unfair expectations in social interaction and/or are intolerant of others’ reasonable views.^22^ An advantage of this reasonableness standard is that it incorporates epistemic humility; the psychiatrist only has to detect and improve moral views that fall below the threshold of reasonability. This does not require exceptional moral insight and protects against overzealous interventions.

Taken alone, however, the reasonableness threshold is insufficient because changing people’s unreasonable moral values is not necessarily a psychiatric goal. Therefore, the psychiatrist should only aim to change patients’ unreasonable moral views when it is instrumental for achieving psychiatric goals.iii In the case of the ex-enforcer, the psychiatrist is justifiably concerned that the patient’s moral views increase the risk of relapse.

Although moral argument entails treating the patient as an autonomous agent that can respond to reasons, the substantive approach does bring some risk of coercively changing the patient’s views because, for example, the psychiatrist is (rightly) seen as an authority that can exert power over the patient. However, coercive changes to the patient’s moral views are likely to be less robust than changes based on reasons the patient appreciates. Therefore, the psychiatrist should take care to ensure that any substantive moral changes the patient makes have been made autonomously since this both respects patient autonomy and will tend to be more effective.

The substantive approach does have the potential to damage the therapeutic relationship. Obviously, any effort to change the patient’s mind about a moral issue has to be broached carefully since it will be self-defeating if the therapeutic relationship breaks down. The patient might experience a direct challenge to the morality of their beliefs and actions as a sign of disrespect or coercion; however, efforts of persuasion need not be confrontational. The psychiatrist might, for example, find ways to cast an alternative moral position in a good light while appearing to remain neutral. In any case, the risk of damaging the therapeutic relationship is worth taking when the patient’s unreasonable moral views are a barrier to therapeutic progress; to *not* address those views would be even more certainly self-defeating. It is worth noting that taking the substantive approach does not entail blaming the patient and the serious damage to the therapeutic relationship that blame would cause. Judgements about patients’ moral capacities and beliefs do not entail judgments of *blameworthiness* or condemnation.^23^ Here, the attitude of ‘detached concern’ may be especially useful.^24^ In detached concern, one maintains some emotional distance (eg, tendencies to blame are limited) while remaining sensitive to the objective features of the patient’s plight, including, on our view, the moral features.

The substantive approach is also justified in some cases where patients have underdeveloped moral conceptions. Take the following example:

A woman with autism is struggling to develop close relationships outside her immediate family but she doesn’t know why. She wants guidance on how to navigate the ethical norms in relationship building. In therapy, it becomes clear that she is indifferent to others’ interests and doesn’t see why she should show interest in things that initially strike her as boring.

In this case, the patient needs some novel content for her conception of morality, so the procedural approach is at a loss. On the substantive approach, however, the psychiatrist can inform her that in close relationships, there are additional moral expectations, such as being open to shaping each other’s interests.^25^ By providing this extra content (or something like it), the psychiatrist supports the patient’s autonomy and helps her flourish.

Helping people elaborate a conception of morality has little risk of coercion and damage to the therapeutic relationship because the patient is requesting that content and is not committed to a conflicting moral position. Of course, it would be wrong to simply indoctrinate the patient so, to avoid that, the process of moral development should be patient-led as much as possible. However, if the moral views the patient gravitates towards in this process are clearly unreasonable, then the psychiatrist has an obligation to guide the patient’s views back within the bounds of reasonableness.

Psychiatrists are well placed to affect substantive moral growth. Their skill for helping people understand and elaborate their subjective worlds can reveal where the moral growth required to treat mental illness and support flourishing might be most easily achieved. We suggest a pluralist approach where the psychiatrist draws on any moral reasons, arguments or insights that help the patient achieve moral growth. In order to tailor moral reasons to the patient, psychiatrists would benefit not only from training in normative moral theories (eg, contractualism, deontology, consequentialism) but also from familiarity with a diverse range of autobiographical or fictional narratives that illustrate how different moral views are experienced and put into practice. The latter would also provide material that the psychiatrist could draw on to help the patient develop moral aspects of their own self-narrative. In the near future, the substantive approach could also benefit from pharmacotherapies, such as psychedelics,^26^ which might help patients who consent to such treatment become more receptive to new moral reasons, beliefs and emotions.

## Conclusion

Psychiatry should take the procedural approach to patient morality by default and only escalate to the substantive approach in cases where the patient has clearly unreasonable or underdeveloped moral views that are relevant to psychiatric outcomes. We do not yet know how effective psychiatry could be in making substantive moral changes; no doubt it will often be challenging and even sometimes futile. Nevertheless, small improvements in morality are worth pursuing because they can have significant benefits to patient and society.

## Data Availability

Data sharing not applicable as no datasets generated and/or analysed for this study.

## Footnotes

Although we focus on psychiatric contexts, much of what we say here could be extended to other psychotherapeutic contexts.

The procedural and substantive approaches share some similarity with the models of physician-patient relationship put forward by Emanuel and Emanuel.^20^ The Interpretive Model, where the physician helps the patient work out their medically relevant values and choose a treatment accordingly, is analogous to the procedural approach. The Deliberative Model, where the physician might persuade the patient to reconsider their medically relevant values, is analogous to the substantive approach. We cannot engage in a thorough analysis of the relationships between these concepts here but a key difference is that, on the procedural and substantive approaches, a goal of treatment is to sharpen or change the patient’s moral outlook, not to help the patient make a decision about treatment.

For those who take a wide view of psychiatry, whereby psychiatric goals are all instances where psychiatric technologies can improve flourishing, then substantive interventions are limited by considerations of distributive justice as mentioned above.
